# Management of Pediatric Mandibular Fracture With Acrylic Cap Splint

**DOI:** 10.7759/cureus.33324

**Published:** 2023-01-03

**Authors:** Himaja Swayampakula, Shreya Colvenkar, Bhuvaneshwari Kalmath, Jayasri Vanapalli, Mohammed Ali Zaheer

**Affiliations:** 1 Department of Oral and Maxillofacial Surgery, MNR Dental College and Hospital, Sangareddy, IND; 2 Department of Prosthodontics, MNR Dental College and Hospital, Sangareddy, IND; 3 Department of Oral Medicine and Radiology, MNR Dental College and Hospital, Sangareddy, IND

**Keywords:** mandible, fracture, circummandibular wiring, pediatric, trauma

## Abstract

Trauma during childhood can have an extreme mental jolt on the minds of growing children. The fundamentals of treatment of jaw fractures vary among children and adults. In children, minimal manipulation of facial skeleton is necessary to rehabilitate the supporting bony framework to pre-trauma condition. The procedure should not only be non-intrusive but also cause minimum malfunction and aesthetic disability. The case report presents the successful management of a seven-year-old boy with mandibular symphysis fracture using an acrylic cap splint retained with circum-mandibular wiring.

## Introduction

Pediatric facial fracture is frequently a daunting injury with a reported incidence of 4-6% of the total. The range is 0.6 to 1.2% which is even less in the age group below five years [1].

The principles of mandibular fracture management vary among children and adults. In children, minimum alteration of facial skeleton is necessary to rehabilitate the supporting bony framework to pre-trauma condition. Fracture treatment is performed without any delay and depends on the nature of fracture, skeletal growth and stage of dentition progression [2].

In non-displaced or minimally displaced fractures treatment is either monitoring the fractured site or closed reduction. Severely dislocated fractures can be managed with open reduction and rigid internal fixation [3]. In some cases, internal fixation with plates and screws is not possible because of the presence of tooth buds within the mandible. The management of such cases can be done with a mandibular acrylic cap splint attached to the teeth using circum-mandibular wiring. This allows maintenance of oral prophylaxis without tooth bud disturbance.

The case report presents the successful management of a seven-year-old boy with mandibular symphysis fracture using an acrylic cap splint together with circum-mandibular wiring.

## Case presentation

A seven-year-old boy presented to the oral and maxillofacial surgery department with a chief complain of difficulty in mouth opening following a fall from a tree. On clinical examination laceration on the chin, an open bite with bleeding from the mouth and deranged occlusion was present. On palpation, tenderness and step deformity were present around the mandibular lower border in the symphysis region. Condylar movements were diminished on the right side.

**Figure 1:**
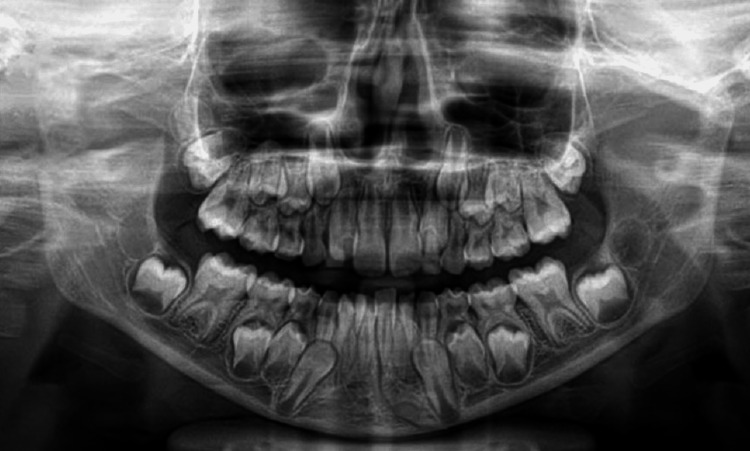
Pretreatment orthopantomogram

Preoperative orthopantomogram (OPG) confirmed symphysis and right condyle fracture (Figure 1).

**Figure 2:**
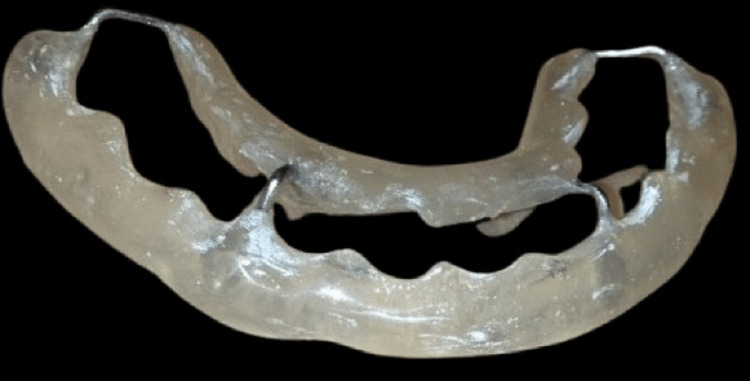
Acrylic cap splint

Under local anesthesia maxillary and mandibular irreversible hydrocolloid impressions were made and casts were poured with dental stone. An acrylic cap splint was fabricated (Figure 2).

**Figure 3:**
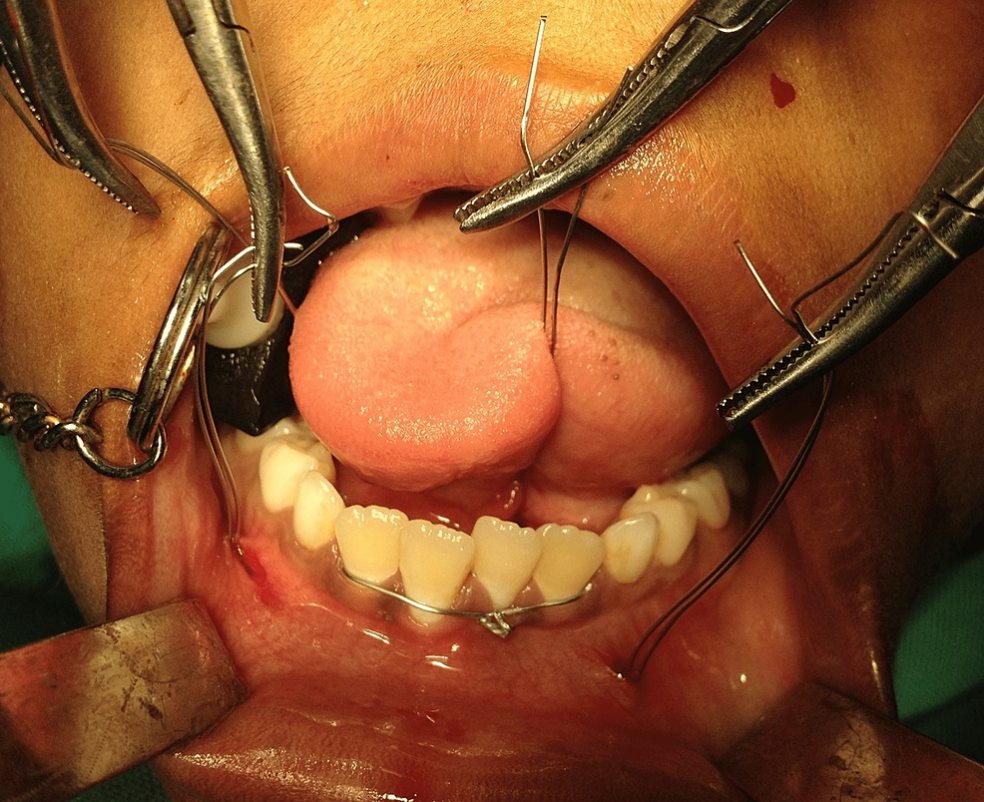
Circum-mandibular wiring

Mandibular symphysis fracture was immobilized with the acrylic cap splint and circum-mandibular wiring. A 26-gauze wire was passed with mandibular awl percutaneously from the existing region. It was clamped in close proximity to alveolus on the lingual side and then on the buccal side once it exited that region (Figure 3).

**Figure 4:**
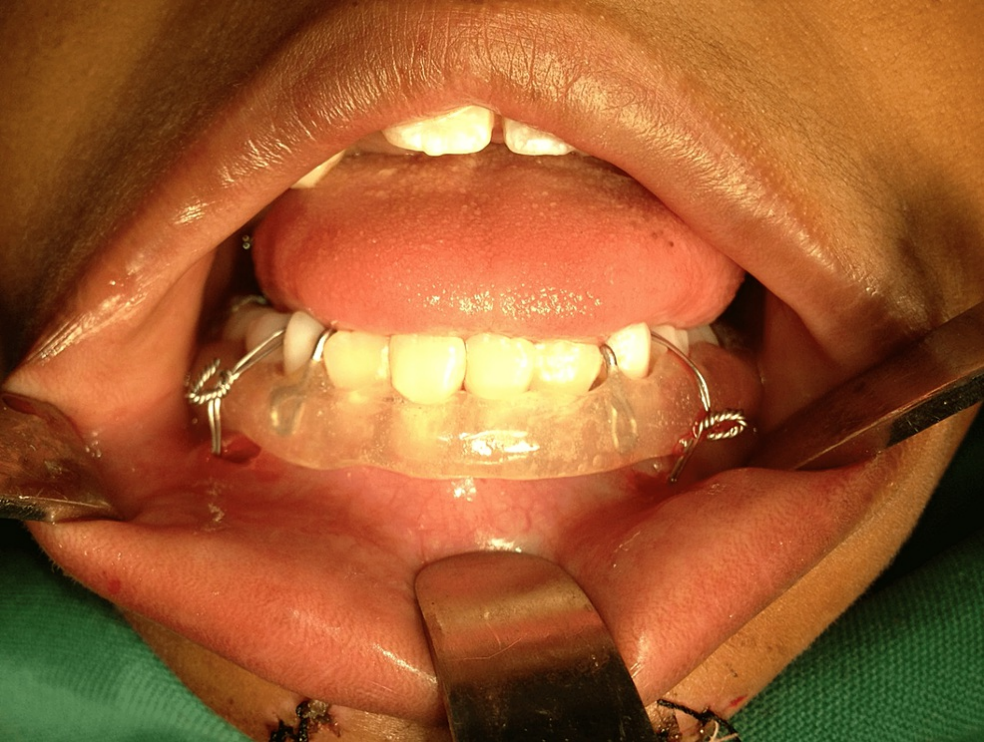
Acrylic splint placed intraorally

The awl and the excess wire cut to the desired length. The splint was held in correct position by twisting and tightening the wires (Figure 4).

**Figure 5:**
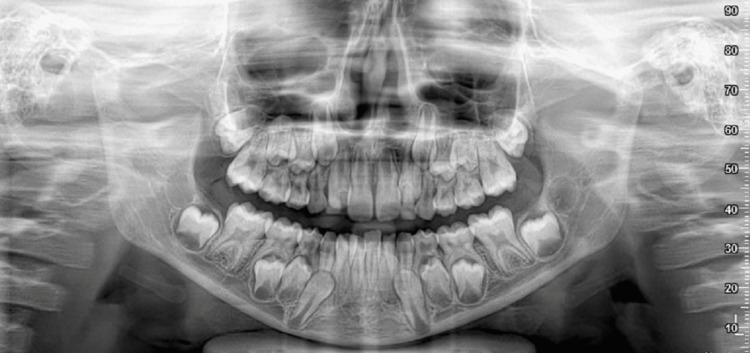
Follow-up orthopantomogram

Postoperative orthopantomogram was taken with acrylic cap splint and circum-mandibular wires in position. The patient was examined every week to understand any other problems faced during the healing phase. The acrylic cap splint and circum-mandibular wiring were removed under conscious sedation during the third week of follow-up. The fractured site showed no signs of mobility. Postoperatively, patients had stable occlusion and complete healing of fractured segments (Figure 5).

The preoperative mouth opening was 10 mm and in the third postoperative week the mouth opening was improved to 28 mm. The patient had excellent occlusion and good chewing capacity during monthly follow-up till six months.

## Discussion

Mandibular fractures are the most common in pediatric patients with male predilection [1]. Pediatric maxillofacial fractures are uncommon and present a unique challenge to the maxillofacial surgeon during management because of potential growth implications. The fundamentals of treatment of fractures of jaws vary among children and adults because of mandibular growth, differences in anatomy, faster recovery, and degree of patient acceptance and cooperation during treatment [4].

Various difficulties are faced during the treatment of mandibular fractures in pediatric patients which include an unstable anchorage system owing to root resorption and deciduous teeth attrition together with uncertain stability in the mixed dentition phase [5]. Primary teeth are not adequately stable and may be avulsed while placing inter-maxillary fixation using arch bars and eyelets. Also, the bony architecture of pediatric patients is soft which makes incompletely erupted teeth unstable [6]. It also results in reduced dietary intake which can cause significant weight and protein loss.

In open reduction, bone plating is done with the help of titanium plates and stainless steel which may adversely affect the tooth bud of permanent teeth. This can cause growth restriction. Recently resorbable plates for open reduction and fixation are used, but poor mechanical properties and the unpredictive nature of resorbability of these plates can hinder the healing process [7,8]. Hence acrylic cap splint was an ideal solution in this case.

The closed reduction with acrylic splint and circum-mandibular wiring provided a positive environment for rapid osteogenesis and prevention of any type of non-fibrous union. It also allowed the pediatric patient to carry on with daily activities because of reduced pain and mobility of the fractured segments.

Acrylic cap splint is a simple and time-honored technique to reestablish occlusion and function during the management of pediatric fractures. It does not interfere with jaw growth and hinders the development of dentition. The acrylic cap splint is easy in terms of insertion and removal and provides maximum stability during the healing phase. It helps in the maintenance of oral hygiene as well as adds to the patient’s comfort. The surgical procedure can be carried out in less time with little or no trauma to the adjoining anatomic structures. The disadvantage is that a specialized technician is needed to carry out the fabrication of the splint. Secondly, the thickness of the splint must be uniform so that it does not interfere with occlusion. Cap splint has limited use in severely displaced fractures and reduced compliance in children since the splint has to be placed in position for at least three weeks.

Hence, this treatment modality is a good option for pediatric patients in mixed dentition stage and thus remains as a novel method for the management of pediatric mandibular fractures.

## Conclusions

Anatomic complexity of the developing mandible may interfere with periosteal envelope resulting in an unstable outcome on development. Hence, closed reduction is the most favorable solution. During Intermaxillary fixation technical problems may arise, hence closed reduction with acrylic splints and circum-mandibular wiring remains the treatment of choice for pediatric patients in the mixed dentition stage. The case report presents the successful management of a seven-year-old boy with a mandibular symphysis fracture using an acrylic cap splint retained with circum-mandibular wiring.
